# Harnessing the Potential of *Bacillus altitudinis* MT422188 for Copper Bioremediation

**DOI:** 10.3389/fmicb.2022.878000

**Published:** 2022-05-19

**Authors:** Maryam Khan, Muhammad Kamran, Roqayah H. Kadi, Mohamed M. Hassan, Abeer Elhakem, Haifa Abdulaziz Sakit ALHaithloul, Mona H. Soliman, Muhammad Zahid Mumtaz, Muhammad Ashraf, Saba Shamim

**Affiliations:** ^1^Institute of Molecular Biology and Biotechnology, The University of Lahore, Lahore, Pakistan; ^2^School of Agriculture, Food and Wine, The University of Adelaide, Adelaide, SA, Australia; ^3^Department of Biology, College of Science, University of Jeddah, Jeddah, Saudi Arabia; ^4^Department of Biology, College of Science, Taif University, Taif, Saudi Arabia; ^5^Department of Biology, College of Sciences and Humanities, Prince Sattam Bin Abdulaziz University, Al-Kharj, Saudi Arabia; ^6^Biology Department, College of Science, Jouf University, Sakaka, Saudi Arabia; ^7^Botany and Microbiology Department, Faculty of Science, Cairo University, Giza, Egypt; ^8^Biology Department, Faculty of Science, Taibah University, Al-Sharm, Yanbu El-Bahr, Saudi Arabia

**Keywords:** copper bioremediation, biosorption, industrial wastewater, isotherm, thermodynamics

## Abstract

The contamination of heavy metals is a cause of environmental concern across the globe, as their increasing levels can pose a significant risk to our natural ecosystems and public health. The present study was aimed to evaluate the ability of a copper (Cu)-resistant bacterium, characterized as *Bacillus altitudinis* MT422188, to remove Cu from contaminated industrial wastewater. Optimum growth was observed at 37°C, pH 7, and 1 mm phosphate, respectively. Effective concentration 50 (EC_50_), minimum inhibitory concentration (MIC), and cross-heavy metal resistance pattern were observed at 5.56 mm, 20 mm, and Ni > Zn > Cr > Pb > Ag > Hg, respectively. Biosorption of Cu by live and dead bacterial cells in its presence and inhibitors 1 and 2 (DNP and DCCD) was suggestive of an ATP-independent efflux system. *B. altitudinis* MT422188 was also able to remove 73 mg/l and 82 mg/l of Cu at 4th and 8th day intervals from wastewater, respectively. The presence of Cu resulted in increased GR (0.004 ± 0.002 Ug^−1^FW), SOD (0.160 ± 0.005 Ug^−1^FW), and POX (0.061 ± 0.004 Ug^−1^FW) activity. Positive motility (swimming, swarming, twitching) and chemotactic behavior demonstrated Cu as a chemoattractant for the cells. Metallothionein (MT) expression in the presence of Cu was also observed by SDS-PAGE. Adsorption isotherm and pseudo-kinetic-order studies suggested Cu biosorption to follow Freundlich isotherm as well as second-order kinetic model, respectively. Thermodynamic parameters such as Gibbs free energy (∆G°), change in enthalpy (∆H° = 10.431 kJ/mol), and entropy (∆S° = 0.0006 kJ/mol/K) depicted the biosorption process to a feasible, endothermic reaction. Fourier Transform Infrared Spectroscopy (FTIR), Scanning Electron Microscopy (SEM), and Energy-Dispersive X-Ray Spectroscopy (EDX) analyses revealed the physiochemical and morphological changes in the bacterial cell after biosorption, indicating interaction of Cu ions with its functional groups. Therefore, these features suggest the potentially effective role of *B. altitudinis* MT422188 in Cu bioremediation.

## Introduction

Heavy metals are one of the most persistent pollutants found on Earth due to their non-biodegradability and tendency to accumulate in the environment, causing harm to our natural ecosystems in the process (Gaur et al., 2021). There are many sources of heavy metals which can take root from natural and anthropogenic origins, with the former including soil run-off, volcanic eruptions, weathering, and erosion (Soltani-Gerdefaramarzi et al., 2021), whereas the latter are anthropogenic sources that stem from several industrial processes such as ore mining, combustion of fossil fuels, and the production of alloys, steel, plastic, dyes, and pigments, respectively (Selvi et al., 2019). Like soil and surface resources, water resources too tend to get contaminated by heavy metals, due to their closeness to industries which require large amounts of water for cooling and other processes, which ultimately enhances the precipitation of heavy metal ions onto the water bed once they enter the closest source (Briffa et al., 2020).

Among many heavy metals that are specifically categorized on the basis of their essentiality and non-essentiality to living organisms, copper (Cu) is an essential metal for biological systems as it is required for the function of various significant processes in prokaryotes and eukaryotes (Oves et al., 2016). It is a soft metal which conducts both heat and electricity, and belongs to the group IB in the Periodic Table of elements, in between nickel (Ni) and zinc (Zn). It is naturally found as a mineral, bound with other elements in the form of sulfides, oxides, and carbonates (Crichton and Pierre, 2001). Though found in two oxidation states in nature, it is usually found in its monovalent form (Cu^+^), which is required by various organisms for essential biological functions, as well as a co-factor for many enzymes, albeit in low concentrations, as elevated levels of Cu tend to induce cellular toxicity and can result in cell death (Zhao et al., 2010). Increasing levels of Cu in our environment have resulted in its pollution, which has since been aggravated due to many anthropogenic and natural factors (Izydorczyk et al., 2021; Covre et al., 2022).

Extensive research for mitigating excessive levels of heavy metals in the environment has brought about several methods for combating environmental pollution, as their accumulation poses a grave danger to sensitive ecosystems of the world (Minnikova et al., 2017). Over the years, many physical and chemical methods of remediation have been developed and critically evaluated, but not all were deemed to be sufficiently efficacious (Sayqal and Ahmed, 2021). The need for cheaper, feasible, and environmentally friendly methods was imperative, due to which the focus shifted toward other forms of adsorbents to remove heavy metals from water (Sulaiman et al., 2021). Biological agents, such as plants, algae, and microorganisms were found to be better suited for heavy metal remediation (Elwakeel et al., 2012; El-Liethy et al., 2018; Elgarahy et al., 2020), but the latter appeared to be more beneficial as they improved metal solubility due to the production of organic acids and/or by modification of soil texture due to the production of polysaccharides (Wang et al., 2017; Alves et al., 2022). Microorganisms such as bacteria are considered to be efficacious agents of bioremediation due to their generally well-known resistance mechanisms, and for their tendency to survive and endure extreme environments (Jain et al., 2011; Saeed et al., 2022). Bacterial consortia of several species such as *Alcaligenes faecalis*, *Staphylococcus aureus*, *Streptococcus lactis*, *Micrococcus luteus*, and *Enterobacter aerogenes* was reported to remove various heavy metals such as copper, cadmium, lead, and zinc from wastewaters (Silambarasan and Abraham, 2013). Furthermore, *Bacillus* sp. have also been well-reported as efficient agents of bioremediation from soil and wastewaters over the years (Joo et al., 2010; Kamika and Momba, 2013; Singh et al., 2014; Wierzba, 2015; Babar et al., 2021). *Bacillus* sp. are considered to be potentially effective in removing heavy metals from contaminated sources of soil and water, based on prior knowledge that Gram-positive bacteria are able to resist a much higher quantity of heavy metals than Gram-negative bacteria (Nwinyi et al., 2014). Furthermore, carboxyl group in the peptidoglycan layers of the bacterial cell wall serve a key role in the uptake of heavy metals, due to which *Bacillus* sp. are potential candidates for the bioremediation of Cu and its compounds (Igiri et al., 2018).

This study aimed to investigate the bioremediation potential of a Cu-resistant bacterium isolated from contaminated industrial wastewaters. The bacterium was also evaluated for its growth characteristics, antioxidative, motility and chemotactic behavior, as well as for the presence of metallothioneins. Fourier Transform Infrared Spectroscopy (FTIR), Scanning Electron Microscopy (SEM), and Energy-Dispersive X-Ray Spectroscopy (EDX) analyses were employed to study physicochemical changes to bacterial cells pre and post Cu biosorption.

## Materials and Methods

### Collection of Samples

The wastewater samples (*n* = 10) were collected from 10 different industrial sites each at Kalashah Kaku and Muridke, which are well-known industrial areas in Sheikhupura, Punjab, Pakistan (geographically located at 31° 72′ 50″ North, 74° 26′ 77″ East and 31° 80′ 95″ North, 74° 25′ 34″ East), respectively. Samples were obtained in sterilized containers, after which some of their physical and chemical characteristics *viz.*, pH, concentration of Cu ions, and temperature were checked and noted.

### Isolation and Purification of Selected Bacterial Strain

All the samples were proceeded on Luria-Bertani (LB) agar medium (tryptone 1%; yeast extract 1%; sodium chloride 0.5%; agar 1.5%). Prior to spreading each sample, Cu (0.1 mm) was added (100 μl) after which incubation was done at 37°C for 24 h. The process was repeated until pure bacterial strains were isolated.

### Determination of Minimum Inhibitory Concentration

The purified cultures were streaked on sterile plates of minimal salt medium (1.0 g/l NH_4_Cl; 0.001 g/l CaCl_2_.H2O, 0.2 g/l MgSO_4_.7H_2_O, 0.5 g/l K_2_HPO_4_, 0.001 g/l FeSO_4_.7H_2_O, 5.0 g/l sodium acetate, 1 g/l yeast extract, final pH adjusted to 7.0; Pattanapipitpaisal et al., 2001), supplemented with different concentrations of Cu (0.1–35 mm). The plates were incubated overnight and the process was repeated until the concentration inhibiting the growth of the most resistant isolate was determined (Shamim, 2014). The isolate was then selected for further study.

### Characterization of Selected Bacterial Strain

The chosen strain was identified by observing several parameters such as colony morphology, Gram staining, and other biochemical tests (Cheesbrough, 2006), after which its molecular characterization was confirmed by 16S rRNA sequencing from Macrogen^®^, South Korea.

### Determination of Cross Heavy Metal Resistance

The resistance to other heavy metals was also investigated by test-tube dilution method. Minimal salt broth medium (supplemented with phosphate and sodium succinate) was prepared and autoclaved in test-tubes, to which 1% of fresh bacterial culture was added in aseptic conditions. Variable concentrations of metal ions were added until the concentration at which the bacterial growth was inhibited for every metal was obtained (Shamim, 2014).

### Determination of Optimal Growth Parameters

The optimal conditions for growth parameters such as temperature, pH, and phosphate concentration were investigated with regard to the chosen bacterial strain. Fresh bacterial cells (1%) in sterile broth media were incubated overnight at varying temperatures (10, 15, 20, 25, 37, 40, 40, 50, 55°C). For the determination of pH, various pH values (5–9) were adjusted for each set, whereas for optimal phosphate concentration, freshly inoculated cells were supplemented with phosphate concentrations ranging from 0.1–5.0 mm prior to their incubation at 37°C. Growth of bacterial cells was observed (OD_578_nm) after successful overnight incubation (Shamim and Rehman, 2012).

### Determination of EC_50_ and Effect of Cu on Bacterial Growth

The half maximal effective concentration (EC_50_) was calculated by administering several concentrations of Cu to fresh bacterial cells (1%) in minimal salt broth medium (pH 7). The samples were incubated at 37°C and optical density was determined after subsequent time periods (OD = 578 nm; Pepi et al., 2008). The growth pattern of cells with and without Cu was also investigated (Shamim et al., 2014). In the experimental setup, Cu (0.1 mm) was supplemented to fresh cultured cells (1%) once they reached logarithmic phase (OD_578_ = 0.3–0.4) while no Cu was added to the control. Growth in each setup was noted at every hour by drawing an aliquot of culture (1 ml) for absorbance at 578 nm.

### Cu Uptake Studies

#### Uptake of Cu by Live Bacterial Cells

Purified bacterial cells (1%) were inoculated in sterile minimal salt medium (pH 7), followed by the addition of Cu (10 mg/l) at logarithmic phase (OD = 578 nm). The flask was placed in a shaking incubator for 30 min to achieve acclimatization phase. In the next step, aliquot (15 ml) was drawn from the setup after regular hourly intervals and was harvested *via* centrifugation for 10 min at 15,000 rpm. Washing of cell pellets was done thrice with EDTA (0.5 M) and Milli-Q^®^ water, while supernatants were collected separately. The water collected from washing was used to analyze the amount of Cu ions adsorbed onto the bacterial surface, while the cell pellets (acid digested; 0.2 N HNO_3_) were evaluated for intracellular accumulation of Cu. All three samples (supernatants, acid digested pellets, water obtained from washing) were then investigated for Cu by atomic absorption spectrometry (AAS) analysis (228.8 nm). The overall % increase and decrease in the concentration of Cu (accumulation, uptake and adsorption) was calculated and presented in the form of graphs (Shamim et al., 2014).

#### Effect of Inhibitors on the Uptake of Cu by Live Bacterial Cells

Three experimental setups were prepared as before. The first set consisted of live bacterial cells, Cu ions (10 mg/l) and 2,4-Dinitrophenol (DNP, Sigma-Aldrich; 1 mm; 1st inhibitor), while the second setup consisted of live cells, Cu ions and N, N-Dicyclohexylcarbodiimide (DCCD, Sigma-Aldrich; 100 μm; 2nd inhibitor). DNP acts as an uncoupler of the mitochondrial membrane by blocking electron transport, while DCCD is a metabolic inhibitor which blocks ATPase and modifies the carboxyl group. In the third setup, the uptake of Cu ions was evaluated in the presence of both inhibitors (Shamim et al., 2014). All three setups were analyzed by AAS analysis (228.8 nm).

#### Uptake of Cu by Dead Bacterial Cells

Upon achieving log phase, live cells in the medium were killed by autoclaving. In the next step, Cu was added in the medium (10 mg/l), followed by incubation, growth determination and AAS analysis at hourly intervals as before (Shamim et al., 2014).

### Cu Removal Efficacy by Pilot-Scale Study

The efficacy of Cu removal by bacterial cells was evaluated by pilot-scale study. Regular tap water (10 l), and industrial effluents (including Cu; 10 l) were taken in one and two large-sized plastic containers, respectively, to which bacterial culture (1.5 l) was then added to tap water, and one of the effluent containers, respectively. Cu (10 mg/l) was then added to all three containers. Samples were then harvested (15,000 rpm; 10 min) after 4 and 8 day intervals, after which the supernatants were used to evaluate the percentage (%) removal of Cu (Shamim and Rehman, 2012).

### Estimation of Antioxidative Enzymes

For the preparation of enzyme extract, inoculation of fresh bacterial cells in medium was performed, followed by incubation at 37°C for 24 h. The next day, Cu (10 mg/l) was added to the medium which was then incubated again overnight. Control was run parallel to the experimental setup, where no Cu was introduced to the bacterial cells. After sufficient growth, bacterial cells were harvested and extraction buffer [50 mm NaH_2_PO_4_ (pH 7.5); 1% PVP; 0.5% Triton X-100; 1 mm EDTA] was added to obtained pellet under low temperature (4°C), after the suspension was centrifuged again (Shamim et al., 2014). The supernatant was then accumulated in a sterile falcon tube and was labelled as the enzyme extract, which was then utilized to evaluate the expression of various antioxidative enzymes such as glutathione reductase (GR; Rao et al., 1996), peroxidase (POX; Reuveni et al., 1992), superoxide dismutase (SOD; Beauchamp and Fridovich, 1971), catalase (CAT; Luck, 1965), and ascorbate peroxidase (APOX; Nakano and Asada, 1987).

### Metallothionein Profiling

The profiling of metallothioneins was carried out using SDS-PAGE. Bacterial cells (1%) supplemented with Cu (0.1 mm) as well as control were incubated overnight. After 24 h, pellets were obtained by centrifuging cells in the medium. The supernatant was discarded and the pellet obtained was dried thoroughly and homogenized in 1X gel-loading buffer. Protein marker and bacterial samples were loaded into resolving gel (12%) and stacking gel (5%) and the electrophoresis apparatus (Bio-Rad, United States) was supplied with electrical current from the power supply. The protein bands were subsequently visualized by using staining and de-staining solutions (Laemmli, 1970). The quantitative estimation of protein was also performed using Bradford assay (Bradford, 1976).

### Evaluation of Bacterial Motility and Chemotaxis in the Presence of Cu

Three patterns of bacterial motility (swimming, swarming, and twitching) were evaluated in the presence and absence of Cu on 0.1, 0.3, and 1.0% of agar, respectively (Murray et al., 2010). Bacterial chemotaxis with and without the supplementation of Cu was also evaluated according to Adler (1973).

### Cu Adsorption Isotherm and Kinetic Studies

#### Isotherm Models

Two isotherm models (Langmuir and Freundlich) were used to determine the suitable mode of biosorption. The Langmuir adsorption isotherm model assumes the biosorption occurs as a monolayer, and was calculated using the following formulae (Wierzba, 2015):


(1) $$\frac{Ce}{q_{e}}=\frac{Ce}{q_{\max}}+\frac{1}{\;bq_{\max}}$$

where *C*_e_, *q*_e_ is the amount of metal ions in solution and adsorbed at equilibrium state, respectively, whereas *q*_max_, and *b* denotes the Langmuir and adsorption constant, respectively.

The Freundlich adsorption isotherm model assumes heterogeneous biosorption, with binding taking place as a multi-layer of ions stacked onto bacterial cells, and is denoted by the following formulae (Joo et al., 2010):


(2) $$\ln q_{e}=\ln k_{f}+\frac{1}{n}\;\ln Ce\;$$

where constants of Freundlich isotherm model are represented by *K*_f_ and *n*, respectively.

#### Kinetic Parameters

The kinetic parameters of Cu biosorption were studied by pseudo-first and second-order kinetic models (Lagergren, 1898; Aksu, 2001), using the following formulae:


(3) $$\ln\left(q_{e}-q_{t}\right)=\ln q_{e}-k_{1}t$$

(4) $$\frac{t}{q_{t}}=\frac{1}{k_{2}\left(q_{e}^{2}\right)}+\frac{t}{q_{e}}$$

where *q*_e_ and *q*_t_ represent adsorption of metals ions adsorbed at equilibrium level, and *t*, *k*_1_ and *k*_2_ denote time and constants of first- and second-order kinetics, respectively.

#### Thermodynamic Parameters

Gibbs free energy change (∆G°), enthalpy change (∆H°), and entropy change (∆S°) were calculated using the following formulae:


(5) $$\Delta G{}^{\circ}=-RT\ln K_{D}$$

where the constants *R*, *T* represent universal gas constant, and temperature (Kelvin), respectively.

### Fourier Transform Infrared Spectroscopy Analysis

In order to determine the interaction of bacterial cells with Cu in the medium, FTIR was performed. Samples were prepared by harvesting bacterial cells (24 h) which were previously supplemented with Cu (10 mg/l) as well as control. Samples homogenized in deionized water were then subjected to FTIR analysis in the mid IRF region of 500–4,000/cm^−1^ (Bruker; Deokar et al., 2013).

### Scanning Electron Microscopy and Energy-Dispersive X-Ray Spectroscopy Analysis

Bacterial characteristics before and after Cu biosorption were analyzed by SEM, coupled with EDX. Samples were prepared according to the method of Khan et al. (2016), which were then fixed and dehydrated using fixing agent and alcohol. The samples were then freeze-dried and were coated with gold for SEM-EDX analysis (JEOL, Japan).

### Statistical Analysis

The experimental and control setups were carried out thrice, accompanied with controls in similar experimental conditions. Standard error, mean and standard deviation were estimated using SPSS (IBM; V. 27.0).

## Results

### Purification, Screening and Selection of Cu-Resistant Isolate

In the present study, 25 bacterial isolates were obtained from wastewater samples. From these isolates, the most resistant strain was chosen for its minimum inhibitory concentration, which was demonstrated to be 20 mm for Cu. The selected isolate, labelled as SMK-08, also exhibited resistance to varying concentrations of several other heavy metals. The cross heavy metal resistance pattern was observed in the following order; Ni (25 mm) > Zn (20 mm) > Cr (18 mm) > Pb (12 mm) > Ag (10 mm) > Hg (5 mm).

### Characterization of Cu-Resistant Isolate

The selected bacterial isolate was identified (morphologically and biochemically) and characterized using 16S rRNA ribotyping, which elucidated the isolate SMK-08 as *Bacillus altitudinis*, which was submitted to GenBank under the accession number MT422188.

### Optimal Growth Parameters, Growth in Presence of Cu and EC_50_

**Figure 1:**
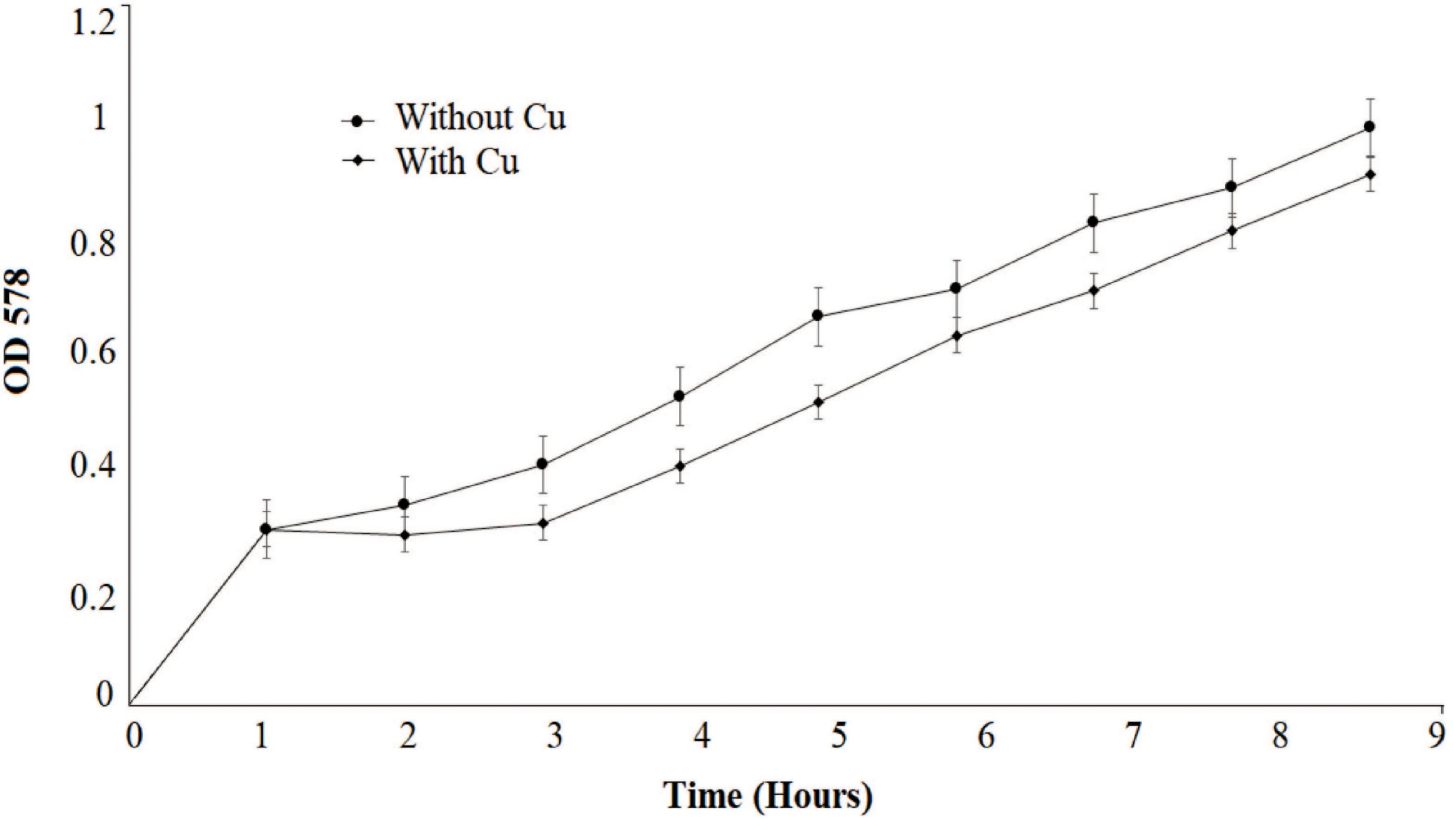
Growth pattern of *Bacillus altitudinis* MT422188 with Cu (0.1 mm) and control.

**Figure 2:**
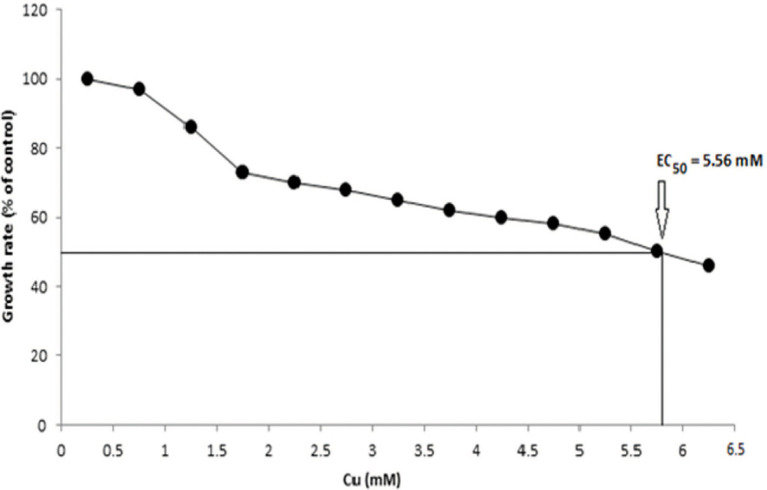
EC_50_ of *B. altitudinis* MT422188 with Cu.

Several growth parameters such as pH, phosphate concentration, and temperature of *B. altitudinis* MT422188 were observed to be pH 7, 1 mm phosphate, and 37°C, which were noted for further experimental setups. The growth curve indicated that the presence of Cu lowered the rate of growth than normal but did not stop it altogether, as shown in Figure 1. The effective concentration (EC_50_) for Cu was observed to be 5.56 mm, depicting that this was the maximal dose at which half the bacterial cells could survive. To validate the results, optical density was obtained (OD = 578 nm) and plotted on the same graph, which demonstrated decline in cellular growth (Figure 2).

### Cu Biosorption Studies

#### Cu Uptake by Live Bacterial Cells

**Figure 3:**
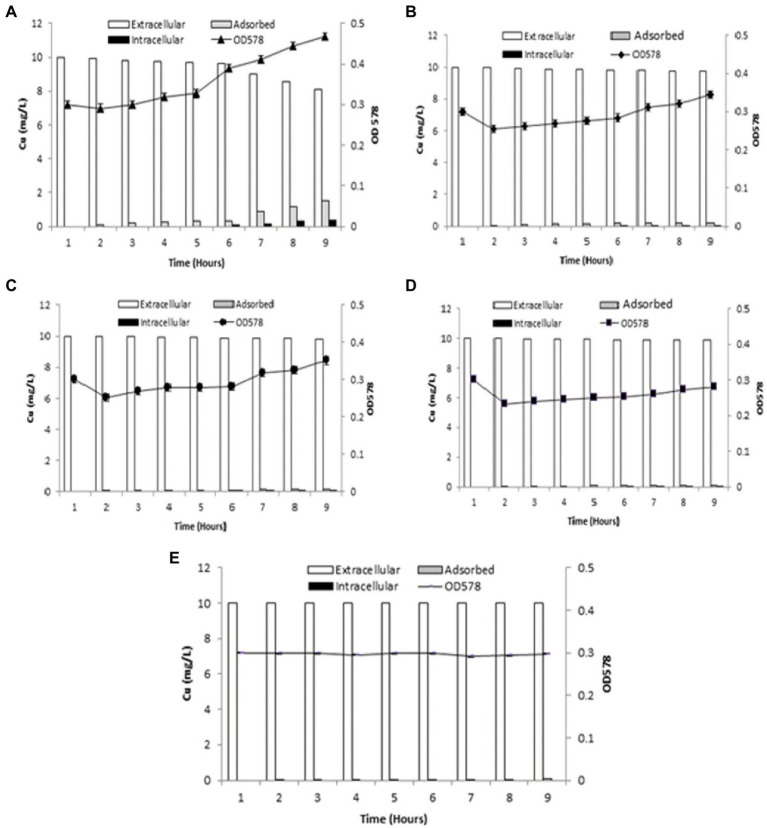
Graphs demonstrating biosorption by *B. altitudinis* MT422188 **(A)** for Cu (10 mg/l; **B**) for Cu (10 mg/l) and DNP (1 mm; **C**) for Cu (10 mg/l) and DCCD (100 μm; **D**) for Cu (10 mg/l), DNP (1 mm) and DCCD (100 μm; **E**) for dead cells and Cu (10 mg/l).

Cu adsorption onto the bacterial surface was observed to be maximum at 9th h (1.52 mg/l), which was also indicated by decrease in Cu present in the medium from 2nd h The intracellular accumulation of Cu increased from 3rd h, with maximum uptake at 9th h (0.37 mg/l). Bacterial cells entered logarithmic phase when added to the medium, whereupon the addition of Cu into aqueous medium, the growth of cells slowly increased from 1st to 5th h, with sharp increase from the 6th to 9th h (Figure 3A).

#### Cu Uptake by Live Bacterial Cells in the Presence of Inhibitors

When inhibitor 1 (DNP) was added to the medium in the presence of Cu, the amount of extracellular Cu in the medium decreased from the original concentration (10 mg/l) in the 1st h to 9.75 ± 0.05 mg/l in the 9th h, which was suggestive of Cu uptake by bacterial cells. The adsorption of Cu onto bacterial cell surface started in the 3rd h (0.1 mg/l) but continued to increase slowly till 9th h (0.19 mg/l). Intracellular accumulation of Cu commenced from 6th h (0.01 mg/l), and continued to increase at regular intervals till 9th h (0.05 mg/l). The growth of bacterial cells dipped between 1st and 2nd h due to acclimatation, but an overall increase was observed after cells entered logarithmic phase (Figure 3B). In the case of inhibitor 2 (DCCD), once the bacterial cells were introduced in the medium, growth dipped at 2nd h, but sharply increased at 7th h Cu adsorption started at 3rd h (0.05 mg/l) which increased at regular intervals up until 9th h (0.16 mg/l). Intracellular accumulation of Cu commenced from 6th (0.01 mg/l) to 9th h (0.05 mg/l), which was due to the action of DCCD and resulted in the slow but active uptake of Cu from the medium (Figure 3C). In the presence of Cu and both inhibitors in the same experimental setup, the adsorption and intracellular accumulation were found to be decreased than the other sets, but the cells were still regulating uptake of Cu, starting at the 6th h till 9th h (0.02 mg/l). Cu adsorption also started at 2nd h, with only 0.02 mg/l adsorbed at 9th h (Figure 3D).

#### Cu Uptake by Dead Bacterial Cells

The dead bacterial cells were not able to grow exponentially in the growth medium as they were inactivated *via* heat. Due to no active uptake process, most of the metal remained in the medium, but the surface of dead bacterial cells provided enough surface area for adsorption onto bacterial cells, which started in 2nd h (0.01 mg/l) and continued till 9th h (0.06 mg/l; Figure 3E).

#### Cu Removal Efficacy by Pilot-Scale Study

**Figure 4:**
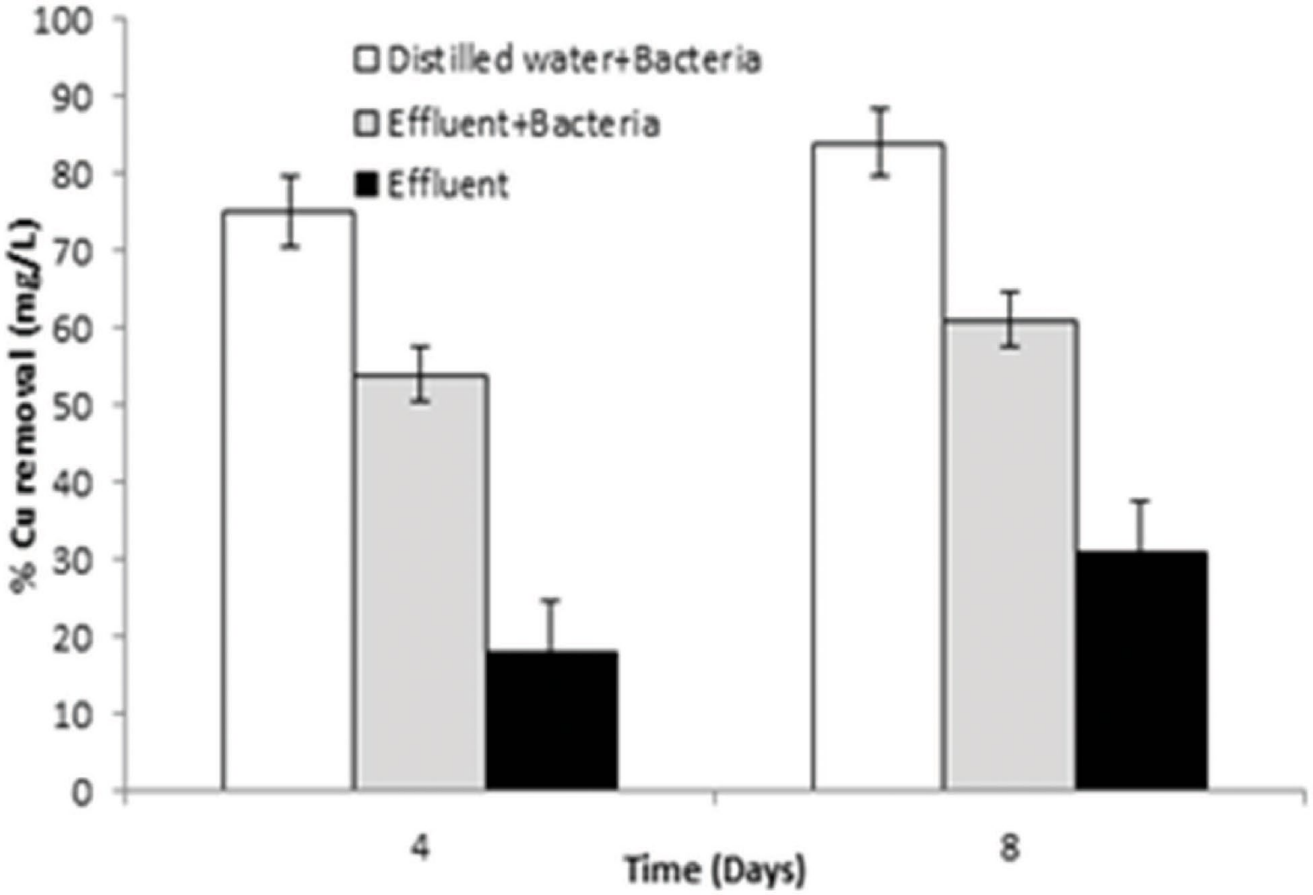
Graph of pilot scale study for biosorption of Cu (10 mg/l) by *B. altitudinis* MT422188 after 4 and 8 days, respectively.

*Bacillus altitudinis* MT422188 cells was able to remove 73 mg/l and 82 mg/l at 4 and 8 day intervals, respectively, from distilled water which contained Cu, whereas the bacterial cells removed 52 and 68 mg/l at day 4 and 8, respectively, from industrial effluent. On the other hand, the original microbial flora of the industrial effluent removed 18 and 28 mg/l at day 4 and 8, respectively, demonstrating that *B. altitudinis* MT422188 is more efficient in removing Cu, as shown in Figure 4.

### Estimation of Antioxidative Enzymes

In *B. altitudinis* MT422188, expression of antioxidative enzymes with and without Cu yielded the increased expression of GR, SOD, and POX. CAT and APOX activity were not induced in either the control or experimental setup (Table 1).

**Table 1:** Expression of antioxidative enzymes of *Bacillus altitudinis* MT422188 with and without Cu.

Experiment	GR (Ug^−1^FW)	POX (Ug^−1^FW)	SOD (Ug^−1^FW)	APOX (Ug^−1^FW)	CAT (Ug^−1^FW)
*B. altitudinis* with Cu	0.004 ± 0.002	0.061 ± 0.004	0.160 ± 0.005	–	–
*B. altitudinis* without Cu	0.001 ± 0.003	0.003 ± 0.001	0.091 ± 0.003	–	–

### Metallothionein Profiling by SDS-PAGE

**Figure 5:**
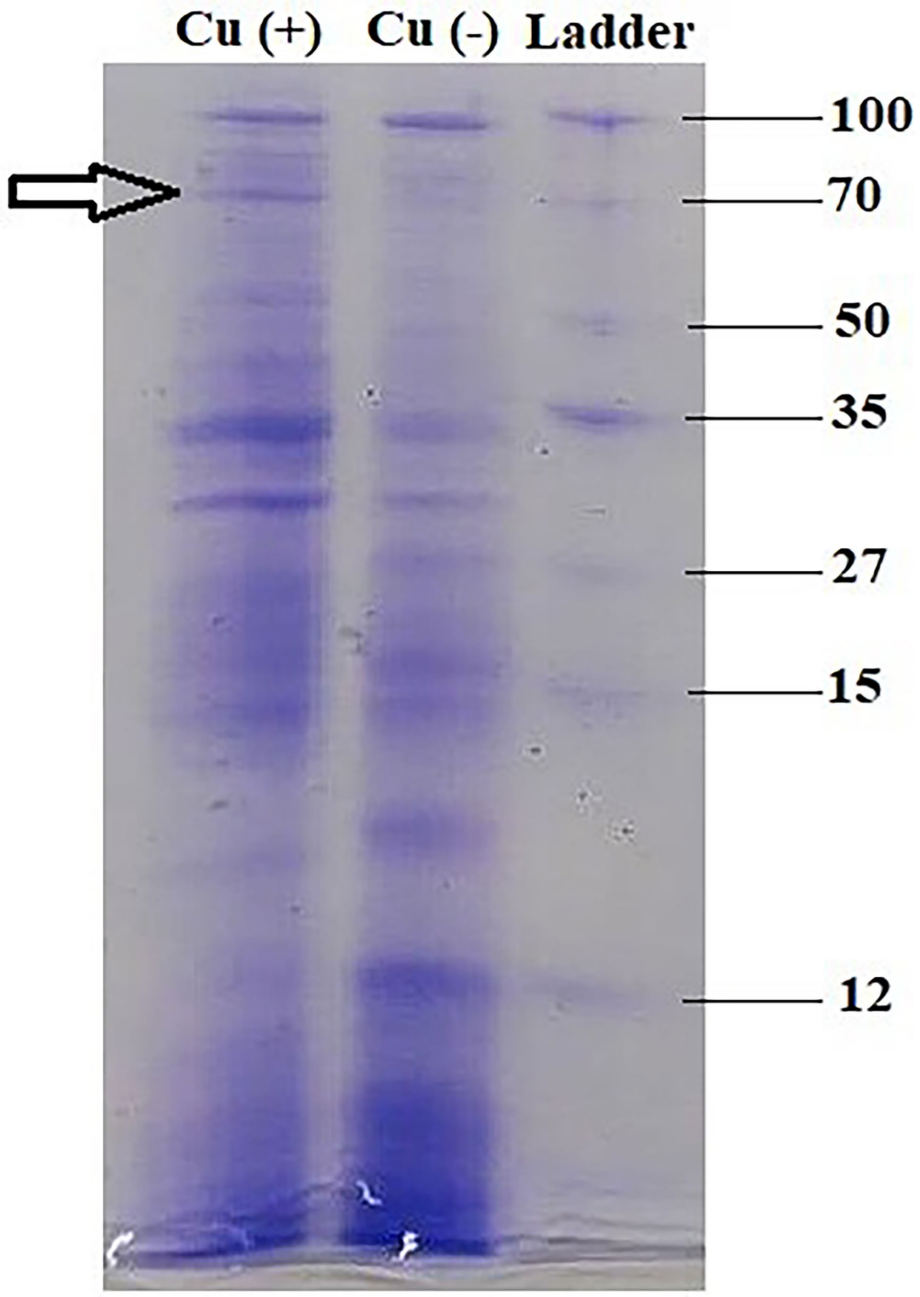
Metallothionein profiling of *B. altitudinis* MT422188 *via* SDS-PAGE.

In bacterial sample (control) without Cu, the induction of several proteins was observed (100, 51, 35, 30, 27, 15, 14, and 12 kDa), whereas in the experimental sample, the presence of Cu elucidated protein of molecular weight 70 kDa among other proteins, suggesting the presence of metallothionein (Figure 5).

### Bacterial Motility and Chemotaxis in the Presence of Cu

**Figure 6:**
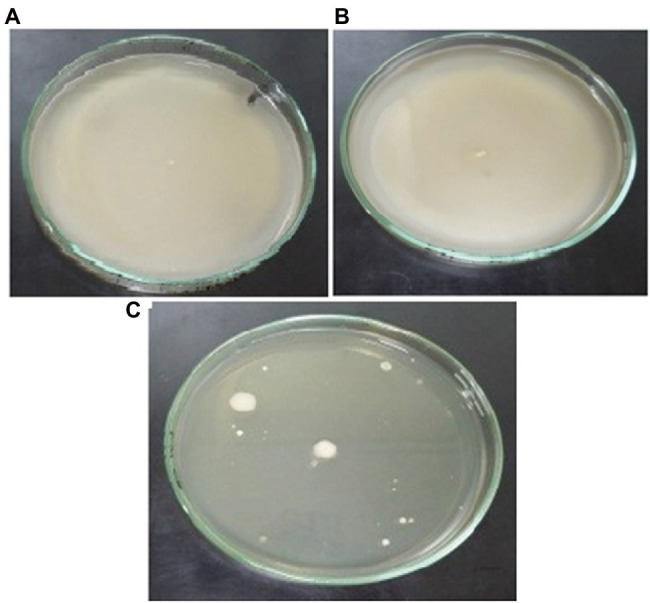
Motility patterns of **(A)** swimming, **(B)** swarming, and **(C)** twitching behavior of *B. altitudinis* MT422188 with Cu (10 mg/l).

**Figure 7:**
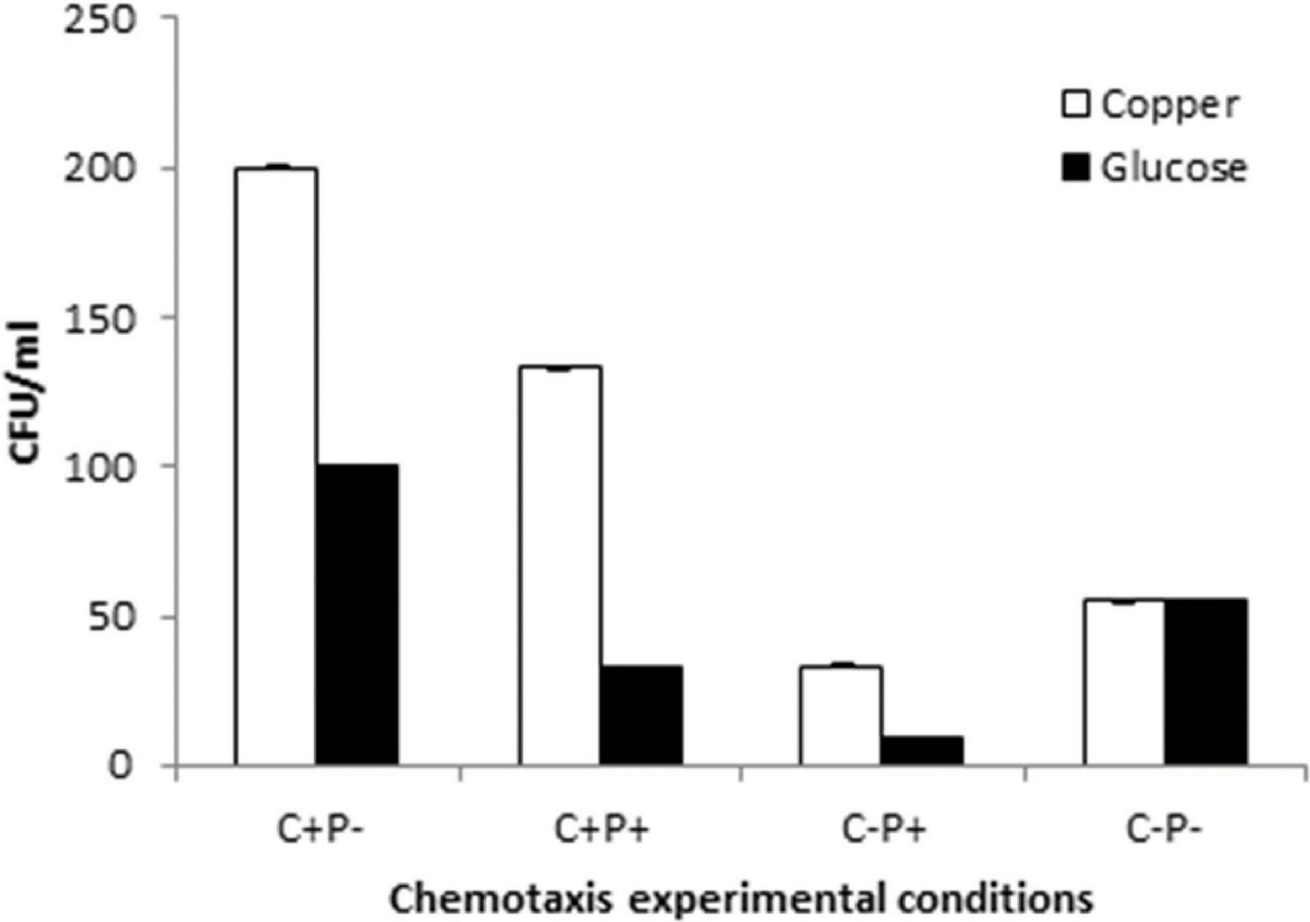
Chemotactic pattern of *B. altitudinis* MT422188 in 4 different setups with Cu and control (glucose). (+) = Cu present, (−) = Cu absent, C = capillary, and P = pond.

The motility experiments demonstrated remarkable patterns of bacterial movement. Swimming and swarming pattern enabled the bacterial cells to cover the entire agar plate, indicating the cells moved freely in the presence of Cu (Figures 6A,B). Twitching motility, however, was less pronounced (Figure 6C). Chemotaxis experiments demonstrated that Cu acted as a chemoattractant for *B. altitudinis* cells, which subsequently activates bacterial flagellar movements resulting in motion toward Cu ions (Figure 7).

### Cu Adsorption Isotherm and Kinetic Order Studies

#### Isotherm Models

Two isotherm models were applied for investigating adsorption of Cu ions onto bacterial cells. The correlation coefficient (*R*^2^) values of Langmuir isotherm were in the range of 0.90–0.95, which was less than the *R*^2^ values of the Freundlich isotherm (0.955–0.999; Table 2), which is why the latter was the suitable model for our work.

**Table 2:** Cu adsorption isotherm model constants of *B. altitudinis* MT422188.

Experiments	Langmuir isotherm			Freundlich isotherm		
	*q_max_*	*b*	*R* ^2^	*n*	*K_f_*	*R* ^2^
Live cells + Cu	4.504	0.0015	0.9515	1.83	1.574	0.9999
Live cells + Cu + DNP	4.699	0.0025	0.9058	1.828	1.545	0.9999
Live cells + Cu + DCCD	3.381	0.0006	0.9206	1.752	1.536	0.997
Live cells + Cu + DNP + DCCD	1.594	0.0005	0.9331	1.75	1.535	0.9996
Dead cells + Cu	9.19	0.0482	0.9237	2.069	0.676	0.9557

#### Kinetic Parameters

The parameters of pseudo-first- and second-order kinetics are given in Table 3. Graphs for first-order were plotted with ln (qe-qt) and qt on the *y*- and *x*-axis, respectively. Correlation coefficients (*R*^2^) for first-order were in the range of 0.9993–0.997, but the values of the experimental and calculated coefficient (*q_exp_* and *q_cal_*) were not in tandem with the values of the equilibrium constant (*q_e_*). In the case of pseudo-second order, the *R*^2^ values were in the range of 0.98–0.99, and the equilibrium constants were also in agreement to the experimental and calculated constants, which therefore implicated the pseudo-second-order to be better fitted for our study (Table 3).

**Table 3:** Pseudo-order (first and second) kinetic model constants for *B. altitudinis* MT422188 biosorption.

Experiments	Pseudo first-order				Pseudo second-order		
	*q_exp_*	*k_1_*	*q_cal_*	*R* ^2^	*k_2_*	*q_cal_*	*R* ^2^
Live cells + Cu	9.11	0.052	2.612	0.9995	0.434	8.438	0.989
Live cells + Cu + DNP	8.88	0.051	2.581	0.9936	0.669	10.44	0.9883
Live cells + Cu + DCCD	8.57	0.0512	2.542	0.9999	1.836	9.8619	0.9974
Live cells + Cu + DNP + DCCD	8.14	0.0535	2.485	0.9998	0.658	10.537	0.9884
Dead cells + Cu	2.11	0.2141	1.383	0.9978	0.5594	10.706	0.9881

#### Thermodynamic Parameters

Gibbs free energy (∆G°) for 10, 25, 37, and 45°C was calculated to be −9.3719, −9.64062, −9.6609, and −4.1655 kJ/mol, respectively. The entropy change (∆S°) was demonstrated to be 0.0006 kJ/mol/K, while the enthalpy change (∆H°) was observed to be 10.431 kJ/mol (Table 4).

**Table 4:** Thermodynamic parameters for *B. altitudinis* MT422188 biosorption.

Thermodynamic parameters			∆G° (kJ/mol)			
	∆H° (kJ/mol)	∆S°(kJ/mol/K)	10°C (283.15 K)	25°C (298.15 K)	37°C (310.15 K)	45°C (318.15 K)
Values	10.431	0.0006	−9.3719	−9.64062	−9.6609	−4.1655

### Fourier Transform Infrared Spectroscopy Analysis

The FTIR peaks elucidated several functional groups which interacted with Cu ions. The peak of 1637.22 cm^−1^ demonstrated C‖O stretching of amide I, as well as the C‖O peptide bond of carboxyl groups, whereas peaks of 3279.39 cm^−1^ was characteristic of hydroxyl, amine, and carboxyl groups in the control, which was determined with the help of peak shifts in experimental and control samples.

### Scanning Electron Microscopy and Energy-Dispersive X-Ray Spectroscopy Analysis

**Figure 8:**
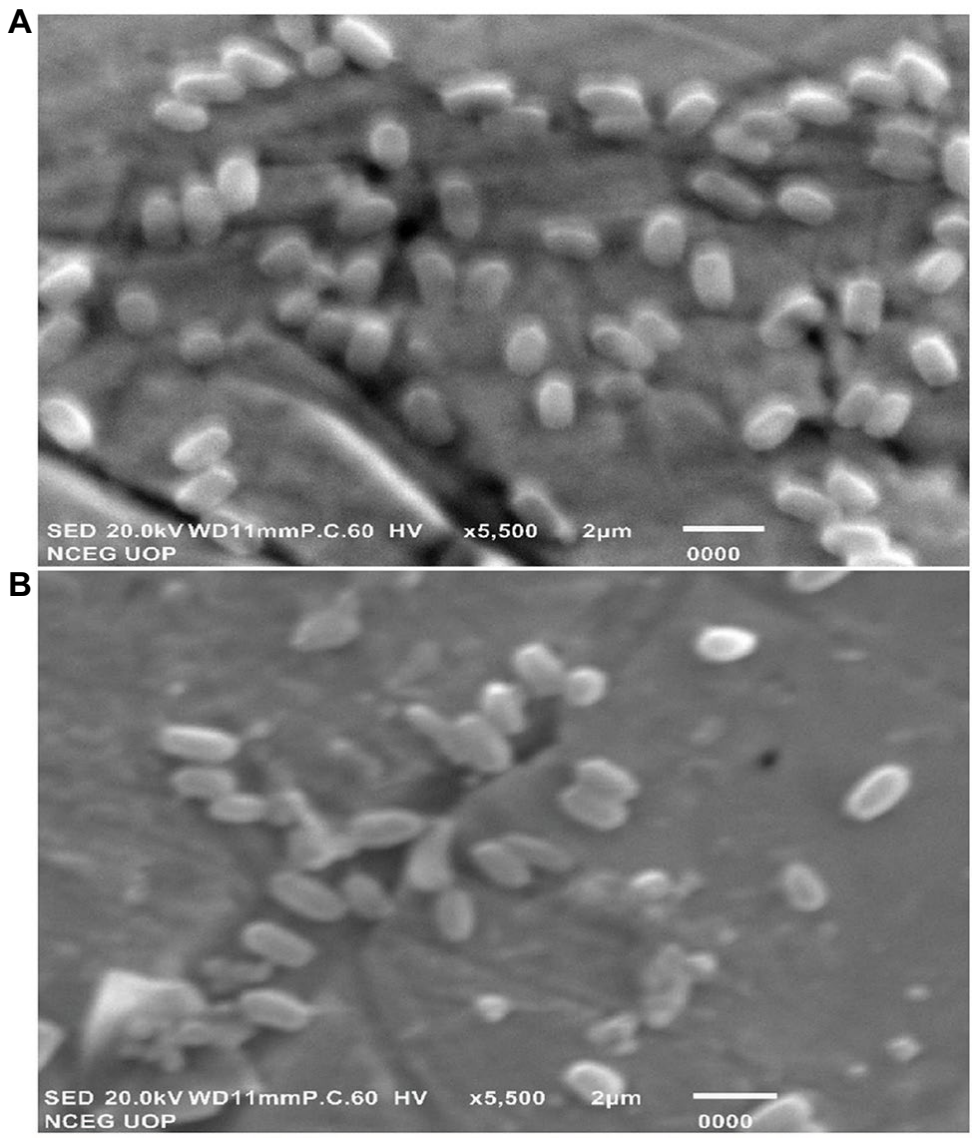
SEM micrographs of *B. altitudinis* MT422188 **(A)** with **(B)** without Cu.

**Figure 9:**
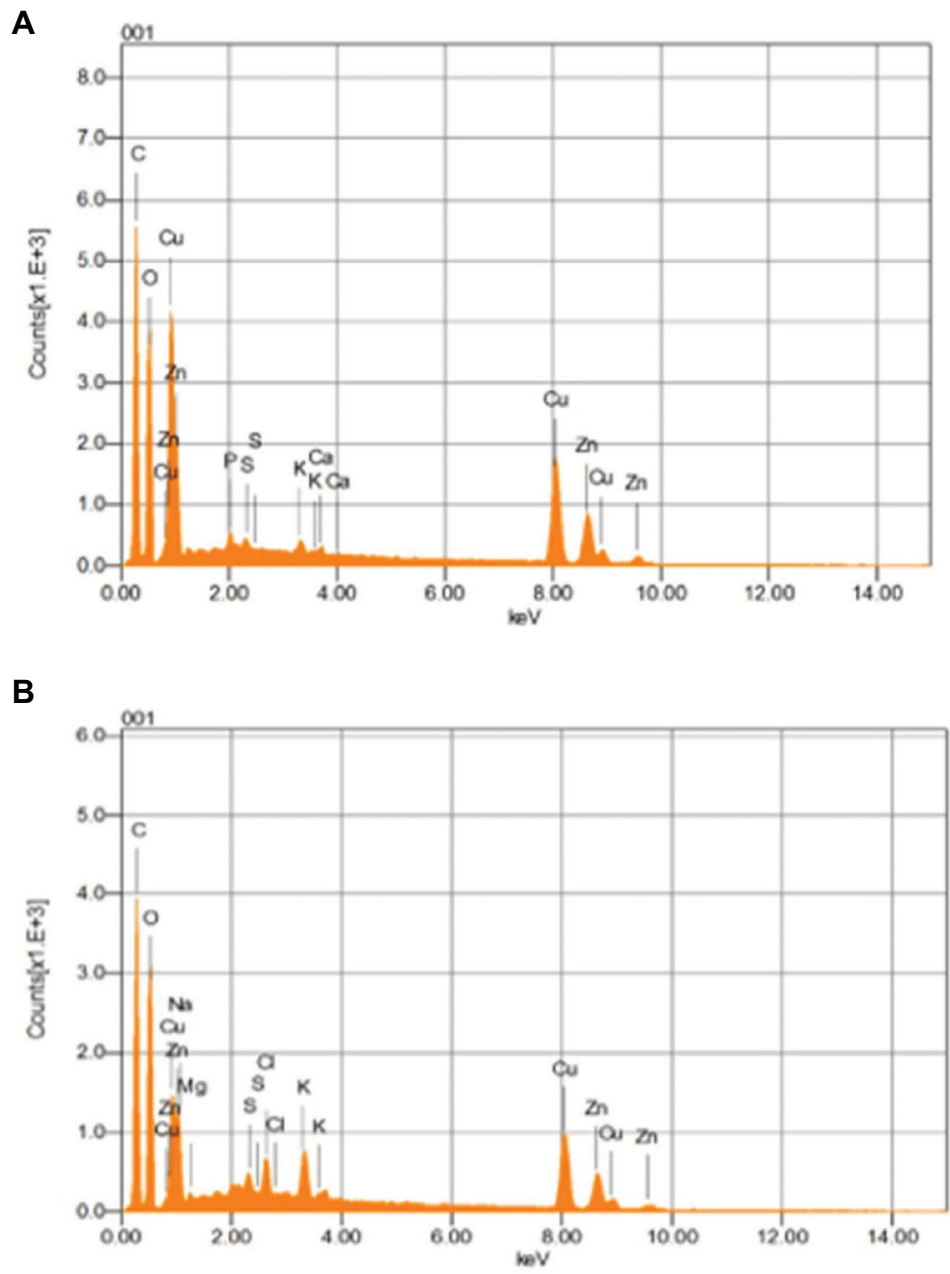
EDX images of *B. altitudinis* MT422188 in **(A)** with **(B)** without Cu.

SEM analysis represented changes in the bacterial cellular morphology and shape after the addition of Cu, where the cells appeared to be a bit flattened and elongated in shape, as compared to bacterial cells which grew without the addition of Cu (Figures 8A,B). EDX analysis represented Cu to be part of the main elements which interacted with the bacterial cell (Figures 9A,B).

## Discussion

The sources of heavy metal contamination in the environment have increased in the past few decades, giving rise to many grave predicaments in nature. It has been a challenge to decontaminate industrial wastewater and soil from heavy metals. Different strategies have been designed and implemented for their removal, including filtration, oxidation/reduction, reverse osmosis and electro-chemical treatment among many others, but are not the favored option due to their cost, inefficiency, and intense labor (Algieri et al., 2021). This research work was carried out to investigate the bioremediation ability of chosen bacterial isolate for removing Cu from contaminated wastewaters. In this study, the chosen strain was identified as *Bacillus altitudinis* MT422188, which was isolated from the industrial areas of Kala Shah Kaku and Muridke, known for their miscellaneous industries and contamination of surrounding areas. It was affirmed in a previous study that a major share of bacteria isolated from heavy metal contaminated environments are Gram positive in nature, and are particularly members of the genus *Bacillus* (Aka and Babalola, 2017).

Cu-resistant bacteria isolated from various sources have been previously reported (Rathi and Nandabalan, 2017; Bhardwaj et al., 2018; Irawati and Tahya, 2021). In our study, the minimum inhibitory concentration (MIC) of *B. altitudinis* MT422188 for Cu was observed to be 20 mm, which was in agreement with the results of Mustapha and Halimoon (2015). The cross metal resistance behavior was also investigated (Ni > Zn > Cr > Pb > Ag > Hg). Rohini and Jayalakshmi (2015) reported the resistance of *Bacillus cereus* against Cu and more heavy metals. *B. subtilis* was reported to be capable of tolerating Cr and Cu upto 20 and 80 mm, respectively (Khusro et al., 2014). *Bacillus* sp. was found to have a high tolerance to many metals, with the most resistance observed against Cu at 12.5 mm, in a study conducted by Esertaş et al. (2020). Similar observations for *Bacillus* sp. were reported in another study (Singh and Chopra, 2014; Gillard et al., 2019). Prior to conducting further experimentation, the growth parameters such as pH, temperature, and phosphate concentration of *B. altitudinis* MT422188 were studied, where it demonstrated favorable growth at pH 7, 37°C, and 1 mm phosphate, respectively. *B. licheniformis* was also reported to demonstrate optimum growth at 37°C and pH 7 (Sher et al., 2021). pH has a pronounced effect on the solubility of heavy metal ions and the charge on the cell surface, which aids greatly in removing the former from contaminated media (Jin et al., 2018), and optimal pH (4–8) is reported to be significantly associated with all biomass forms (Sánchez-Clemente et al., 2020). The growth curves of *B. altitudinis* MT422188 were studied in the presence and absence of Cu, where its presence extended the lag phase of the bacterial cells for a short period of time and did not stop the growth, but resulted in its overall decrease (Figure 1). Similar growth characteristics were observed in *B. licheniformis* in the study of Sher et al. (2021). No effect of Cu on the final growth of *B. stearothermophilus* cells was observed when it was added to medium in a study (Bubela, 1973). In another study, *Bacillus* sp. demonstrated remarkable growth pattern in the absence of Cu, but the overall growth was observed to be decreased in the presence of increasing Cu concentrations (Khan et al., 2017), which was also evident from the findings of our study. Cu ions did not affect growth of *B. thuringiensis* cells greatly, indicating that they survived and grew in low concentrations of the metal (Liu et al., 2020). The half maximal effective concentration (EC_50_) is considered to be the initial concentration of the metal ions at which the activity of the bacterial cells in the presence of metal is comparable to that of 50% of the control culture in the absence of the metal. Due to this aspect, EC_50_ is a determinative parameter that defines the toxicity of metals with respect to bacterial cells. In this study, EC_50_ of *B. altitudinis* for Cu was 5.56 mm, demonstrating that this was the dose at which 50% of the bacterial cells would survive in the presence of Cu (Figure 2). Utgikar et al. (2001) reported the EC_50_ for Cu in their study to be <0.1 mm.

**Figure 10:**
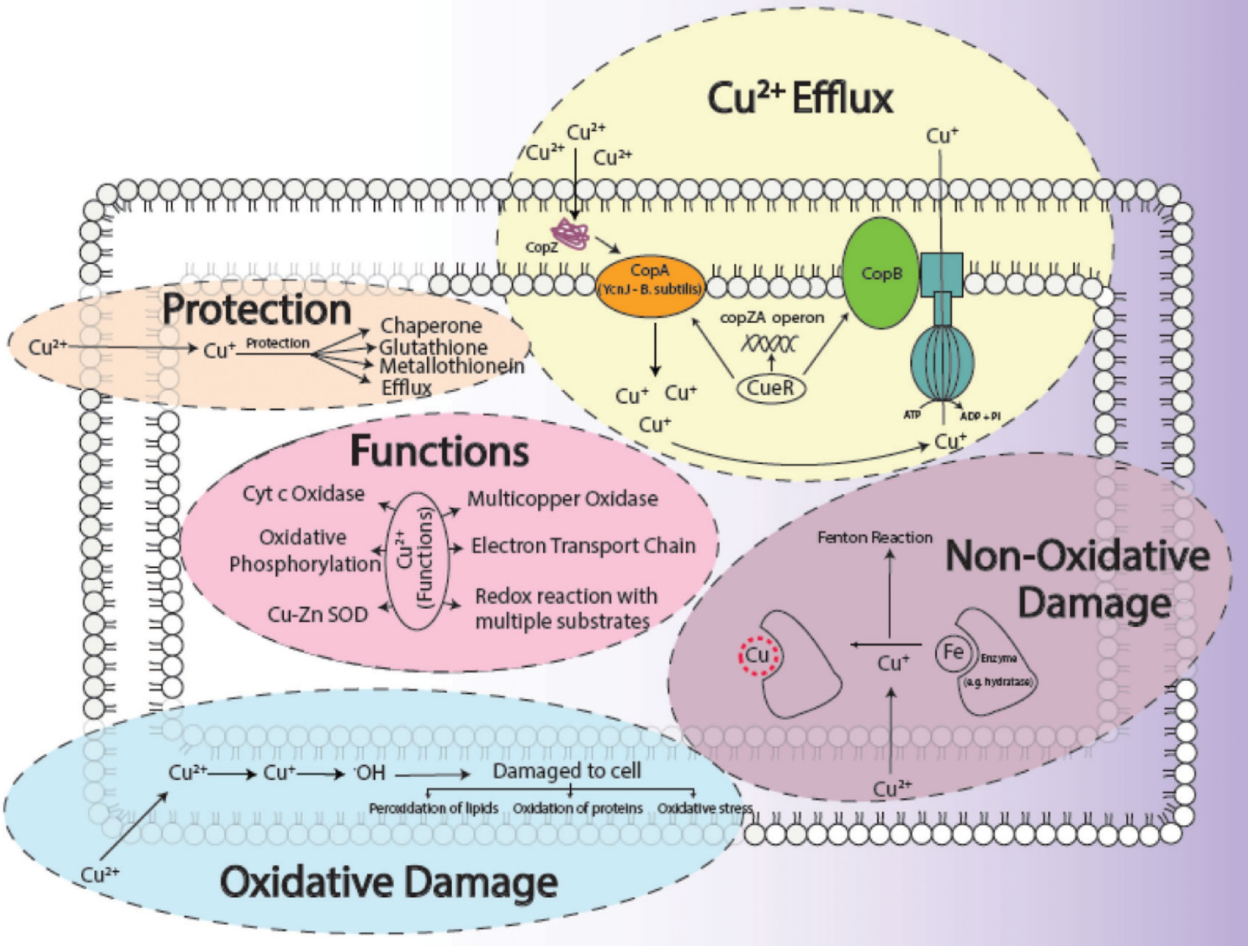
General aspects of copper metabolism in bacterial cells, where it acts as a causative agent for copper-mediated oxidative stress (Ladomersky and Petris, 2015), non-oxidative stress, its efflux (Alotaibi et al., 2021), its major functions (Bondarczuk and Piotrowska-Seget, 2013) in the cell as well as how a cell protects (Giachino and Waldron, 2020) itself from its harmful effects. The conversion of Cu^2+^ to Cu^+^ results in the production of ·OH ions, which damages the cells. The replacement of Fe with Cu^+^ initiates the Fenton reaction which ultimately harms the survival of the cell.

The biosorption studies depicted that in the case of Cu, the amount of extracellular Cu from the medium decreased proportionally from 7th h onwards, particularly due to the increased adsorption of Cu onto the bacterial cells, as well as some of the Cu being accumulated at intracellular level by bacterial cells, indicating that some of Cu was used for the performance of their essential functions. Growth of bacterial cells also noticeably increased after the first 6 h (Figure 3A). When DNP (inhibitor 1) was added to the medium containing Cu and bacterial cells, less pronounced but still eminent uptake was observed as there was some decrease in total Cu present in the medium. Adsorption of Cu also occurred, albeit slowly, from the 3rd to 9th h, while intracellular accumulation of Cu took place in the last 3 h (Figure 3B). DCCD (inhibitor 2) reduced the uptake of Cu from the medium but did not halt it altogether. Adsorption commenced from the 3rd h which continued till 9th h, while the intracellular accumulation was the same as in the case of DNP (Figure 3C). In the presence of both inhibitors and Cu, the bacterial growth rate was reduced. The adsorption and accumulation were also decreased but still occurred nevertheless, indicating that an ATP independent efflux system was employed by *B. altitudinis* MT422188 (Figure 3D). The most commonly reported Cu efflux system in bacterial species is encoded by the *copZA* operon, which is essential for inducing resistance to excessive levels of Cu. CueR is a protein which regulates cytosolic Cu and the operon encoding both Cu chaperones and ATPases for efflux, respectively (Gaballa and Helmann, 2003). CopZ serves the key role as chaperone for delivering Cu to CopA, a CPx-type ATPase, whereas CopB in the operon is responsible for Cu efflux and detoxification (Smaldone and Helmann, 2007; Figure 10).

In many studies, live or dead biosorbents have been used to deduce their efficiency in removing heavy metals from contaminated environments. It has been reported that rendering a bacterial biosorbent inactive by heat (autoclaving) strengthens its efficacy of biosorption (Paul et al., 2012). Therefore, passive uptake seems to be the major mechanism of biosorption in killed cells, as compared to the active uptake in live cells, respectively (Shamim et al., 2014). In this study, the killed cells of *B. altitudinis* MT422188 were able to remove Cu ions from the environment (Figure 3E), the findings of which were consistent with the study findings of Todorova et al. (2019) and Hu et al. (2020). In the pilot scale study, *B. altitudinis* MT422188 was able to remove 73 mg/l and 82 mg/l at 4 and 8 day intervals, which demonstrated its biosorption ability to be efficient in removing Cu (Figure 4)*. B. cereus* has been reported to effectively remove more than 40 and 50% of Cu from industrial effluents (Raj et al., 2018).

**Figure 11:**
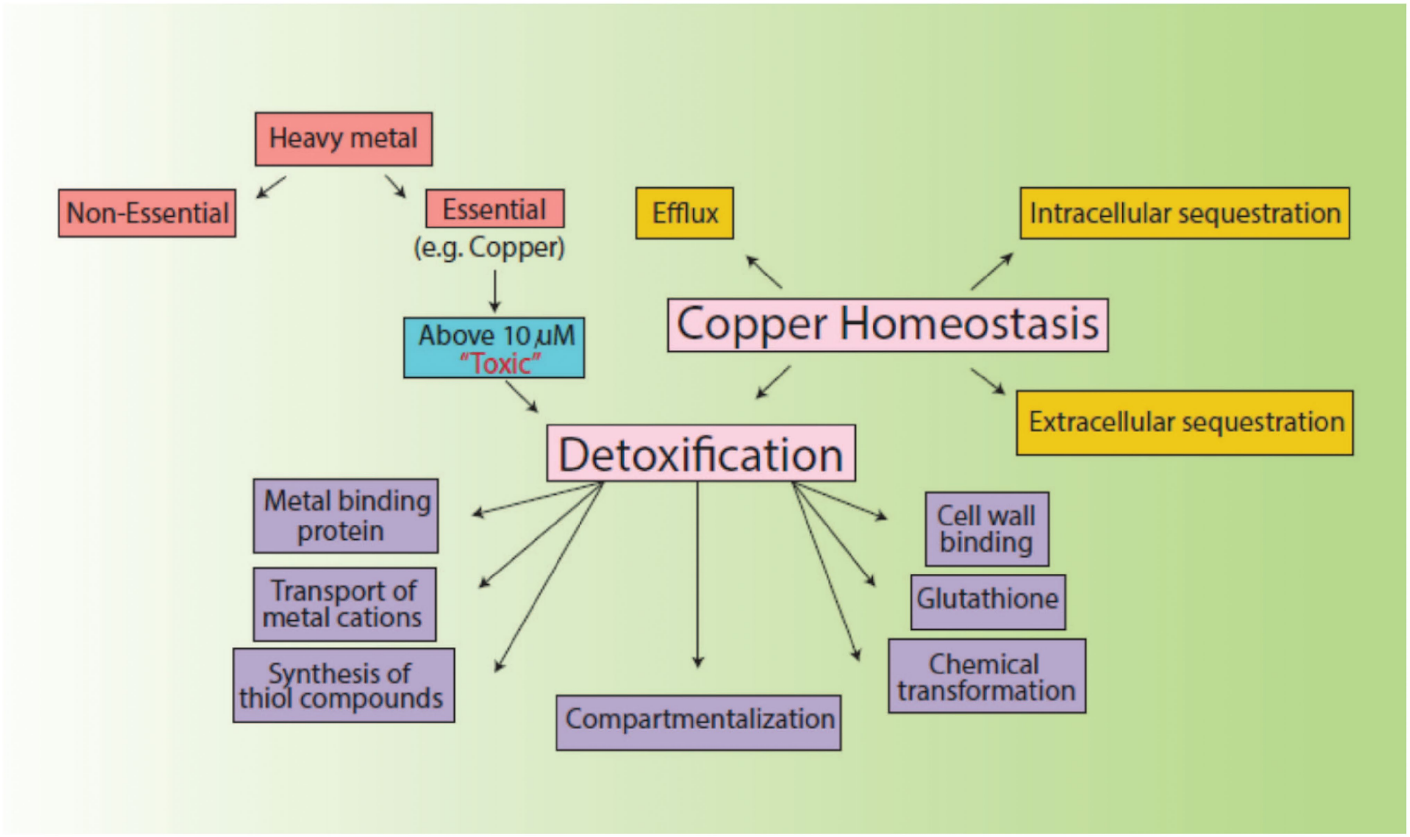
Mechanisms of copper homeostasis and detoxification.

Environmental factors like heavy metal stress can act as key inducers of ROS production, which are directly associated with cell death due to the alteration of intracellular redox stats of the cell (Franco et al., 2009). This increased level of ROS species in the cell can induce a state of “oxidative stress” which can be triggered by toxic concentrations of heavy metal ions and metalloids anions in the cell (Kumagai and Sumi, 2007). In order to combat this, high concentrations of heavy metal ions stimulate the increase in the activity of defense antioxidative enzymes, of which SOD and CAT are the first responders (Choudhary et al., 2007). The presence of divalent or monovalent heavy metal cations, including Cu, can induce the production of ROS through Fenton and Haber-Weiss reactions (Gunther et al., 1995). In our study, the increased activities of SOD, POX, and GR in the presence of Cu was indicative that Cu acted as a stress of *B. altitudinis* MT422188 cells, while CAT and APOX demonstrated no activity (Table 1). High concentrations of heavy metals are reported to trigger oxidative and non-oxidative stress, suggesting increased levels of ROS in metal stressed bacterial cells (Steunou et al., 2020). Cu-Zn SODs were previously thought to be specific to eukaryotes but are now reported to be present in many bacterial species, including *Bacillus* (Argüello et al., 2013). It is pertinent to note that the presence of Cu may have possibly induced the enhanced activity of Cu-Zn SOD in our study. The study findings of Wiesemann et al. (2013) demonstrated elevated SOD activity for mitigating ROS-mediated stress in cells. Another study demonstrated increased CAT activity in the face of elevated H_2_O_2_ stress (Behera et al., 2014), while Latef et al. (2020) reported the elevated POX activity and decreased CAT activity during oxidative stress, respectively. Metallothioneins (MTs) are proteins that are low in weight and abundant in cysteine, which also bind to heavy metal ions with remarkable avidity, and aid in regulating high toxic metal concentrations as well as metal homeostasis (Gadd, 2010). It is reported to be found in many microorganisms including bacteria and fungi, as well as eukaryotes such as plants, animals, and human beings (Chaturvedi et al., 2012). The findings of our study elucidated the presence of a 70 kDa MT protein in *B. altitudinis* MT422188 in the presence of Cu (Figure 5). Another study also reported the role of MT in removing metal ions from the environment *via* sequestration (Mulik et al., 2018). Copper homeostasis involves sequestration, efflux or detoxification as shown in Figure 11. Although essential for the cellular processes, concentrations of Cu^2+^ above 10 μm becomes toxic for the microbial cell. The detoxification mechanisms (Rohini and Jayalakshmi, 2015) are mentioned in Figure 11. During efflux mechanism, CopZ acts as chaperone and converts Cu^2+^ to Cu^+^ which ultimately enters the cells *via* CopA protein. CopB protein is associated with ATPase which is energy dependent and helps in Cu^+^ efflux out of the cell (Alotaibi et al., 2021; Figure 10). To the best of our knowledge, copper sequestration by MTs as well as uptake and efflux mechanisms regulated by copZA operon are apparently involved in copper homeostasis in *B. altitudinis*, as observed in our study.

Motility, particularly in bacteria, is generated by flagellae which enable bacteria to move forward through various movements. There are six different types of movements which cause bacterial motility, such as swimming, swarming, twitching, gliding, sliding, and darting, of which the first two are caused by flagellar movements (Prabhakaran et al., 2016). In our study, swimming, swarming, and twitching patterns of *B. altitudinis* MT422188 were found to be positive in the presence of Cu, which resulted in enhanced motile behavior in bacterial cells (Figures 6A–C). Enhanced motility was also observed in *Bacillus* sp. in the presence of Cu (Singh et al., 2014). In another study, the positive motility and chemotactic behavior of *B. safensis* was reported in the presence of manganese (Mn) ions (Ran et al., 2020). Chemotaxis is a process which involves the regulation of movement away or toward the concentration gradient of specific stimuli or stresses, acting either as attractant and repellent factors, respectively (Vladimirov and Sourjik, 2009). Chemotaxis in bacteria is regulated *via* a chemo-sensory pathway which involves several key proteins which initiate the movement away or toward an external stimulus. In this study, Cu acted as a chemoattractant for the cells, meaning that bacterial cells in solution tended to move toward Cu ions, which initiated flagellar motions of movement in *B. altitudinis* MT422188 (Figure 7).

For the determination of suitable adsorption isotherm, the biosorption data were examined with two mathematical models (Table 2). The study findings indicated biosorption was well-fitted by the Freundlich model (*R*^2^ ≥ 0.98). The Langmuir isotherm model suggests that the process of adsorption is homogenous which occurs at specific sites in the adsorbent, while the Freundlich model suggests the adsorption occurs on a heterogenous surface with varying levels of biosorption affinities. The binding sites with a remarkably stronger binding energy tend to be involved primarily, followed by the exponential decline in the adsorptive energy until the whole process is completed (Pandhare et al., 2013). The Freundlich intensity constant (*n*) is regarded to be independent of the metal ion concentration, where *n* > 1, 1/*n* < 1, 1/*n* > 1 are suggestive of adsorption in favorable conditions, normal adsorption, and co-operative adsorption, respectively. In our study, the value of *n* was found to be greater than 1, which indicated the favorable biosorption of Cu ions. The results were in agreement with other studies which was also reported the Freundlich isotherm model to be well-fitted for *Bacillus* sp. (Liu et al., 2013; Kanamarlapudi and Muddada, 2019). In another study, the same model was also observed to be opted by *Lactobacillus fermentum* for the biosorption of Cu ions (Kargar and Shirazi, 2020). The study of kinetic parameters is important for the examination of the binding processes which can aid in elucidating reaction pathways and their mechanisms of action for subsequent biosorption. In order to determine the kinetic parameters of Cu for *B. altitudinis*, the pseudo-order (first and second) kinetics were investigated (Table 3), which demonstrated pseudo-second-order kinetics to be more suitable for our results than the former. Yang et al. (2017) also described the pseudo-second-order to be well-fitted with their study of Cu biosorption. Change in free energy, enthalpy and entropy (∆G°, ∆H°, and ∆S°, respectively) were calculated in this study in order to evaluate the thermodynamic patterns of Cu biosorption onto *B. altitudinis* cells. ∆G° was observed to be −9.3719, −9.64062, −9.6609, and − 4.1655 kJ/mol, respectively (Table 4). The negative values of free energy change were suggestive of the thermodynamically feasible nature of the biosorption process. The enthalpy change (∆H°) was found to be 10.431 kJ/mol, which indicated that Cu biosorption by *B. altitudinis* MT422188 was an endothermic process. ∆S° demonstrated the reaction to be feasible due to increased randomness at solid–liquid interface, which also agreed with the works of Kaparapu and Prasad (2018) and Sonawdekar and Gupte (2020).

The interaction of Cu with bacterial cell was investigated by FTIR. Peaks after Cu biosorption demonstrated slight shift in various functional groups such as hydroxyl (‖OH) and carboxyl (―C‖O). These peaks were indicative of Cu ion adsorption onto bacterial cells, with the cell membrane and its functional groups playing a significant part in the binding of metal ions, respectively. Many studies reported similar bands of biosorption peaks (Yang et al., 2017; Palanivel et al., 2020). SEM and EDX analyses yielded the physicochemical changes in bacterial cells in the presence of Cu, which was analyzed through SEM micrographs. These changes after Cu biosorption pertained to structural changes in bacterial cells, which were noted to be irregular and more flattened in shape as compared to cells prior Cu biosorption, as shown in Figures 8A,B. Changes in the shape of bacterial cells in response to metal ions has been reported previously by Yang et al. (2017). The changes in bacterial cells in the presence of Cu were also observed by EDX analysis, where sharp, intensified peaks in the range of 0–2 keV and at 8 keV corresponded to the presence of Cu signals in the spectrum, which was less pronounced in the control sample (Figures 9A,B). Our study findings were in agreement with Matilda et al. (2021).

## Conclusion

This research work was conducted to investigate a Cu-resistant bacterium for its potentially efficient ability to remove Cu from polluted wastewater. The selected bacterial isolate SMK-08, identified as *Bacillus altitudinis*, was observed to optimally grow at pH 7, 1 mm phosphate, and 37°C, while its EC_50_ (for Cu) was observed to be 5.56 mm. Biosorption studies indicated the efflux and uptake of Cu in bacterial cells to be an ATP independent process, whereas in the pilot scale study the efficient removal of Cu by *B. altitudinis* MT422188 (73 mg/l and 82 mg/l of Cu at 4 and 8 day intervals) was indicative of its efficacy. Chemotaxis and motility demonstrated positive chemoattractant behavior by *B. altitudinis* MT422188 cells for Cu. GR, SOD, and POX activity in bacterial cells was elevated in the presence of copper, while no expression of CAT and APOX activity was observed in either the presence or absence of Cu. Isotherm study demonstrated the Freundlich model to be the closest fit to the study, while the pseudo-second-order was suited in the kinetics of the study. The thermodynamic parameters depicted the overall process of biosorption to be spontaneous and feasible. FTIR demonstrated interaction of bacterial ―OH and ―C‖O groups with Cu after biosorption, while the SEM and EDX analyses were indicative of the interactions of Cu ions with the bacterial cell wall and other components, resulting in physicochemical changes. Taking these properties presented in our study into consideration, it could be stated that *B. altitudinis* MT422188 is an efficient biosorbent for Cu, and can be employed for its remediation.

## Data Availability Statement

The datasets presented in this study can be found in online repositories. The names of the repository/repositories and accession number(s) can be found in the article/supplementary material.

## Author Contributions

MK performed all the experimentations, writing-review, and editing. MK, RK, MH, AE, and HS performed data analysis, validation, writing–review and editing, and resources. MZ helped in execution and validation of the experimentations. MA facilitated critical review on pre-publication stages. SS conceived and designed the experimental strategies and was the main supervisor of the whole project. All authors contributed to the article and approved the submitted version.

## Funding

This research was funded by Taif University Researchers Supporting Project number (TURSP - 2020/59), Taif University, Taif, Saudi Arabia.

## Conflict of Interest

The authors declare that the research was conducted in the absence of any commercial or financial relationships that could be construed as a potential conflict of interest.

## Publisher’s Note

All claims expressed in this article are solely those of the authors and do not necessarily represent those of their affiliated organizations, or those of the publisher, the editors and the reviewers. Any product that may be evaluated in this article, or claim that may be made by its manufacturer, is not guaranteed or endorsed by the publisher.

## Abbreviations

APOX: Ascorbate Peroxidase
CAT: Catalase
DCCD: N, N-Dicyclohexylcarbodiimide
DNP: 2,4-Dinitrophenol
EDX: Energy-Dispersive X-Ray Spectroscopy
FTIR: Fourier Transform Infrared Spectroscopy
GR: Glutathione Reductase
MIC: Minimum Inhibitory Concentration
MT: Metallothionein
OD: Optical Density
POX: Peroxidase
PVP: Polyvinylpyrrolidone
ROS: Reactive Oxygen Species
rRNA: Ribosomal RNA
SDS-PAGE: Sodium Dodecyl Sulphate-Polyacrylamide Gel Electrophoresis
SEM: Scanning Electron Microscopy
SOD: Superoxide Dismutase
